# Supplementary material for: The health impacts of the COVID-19 pandemic on adults who experience imprisonment globally: A mixed methods systematic review
Source: PLoS One. 2022 May 20;17(5):e0268866. doi: 10.1371/journal.pone.0268866 (PMC9122186; doi:10.1371/journal.pone.0268866)
Supplement: S1 Table — (DOCX) [file pone.0268866.s001.docx]

**S1 Table. Risk of bias for quantitative studies included in a systematic review on the health impacts of the COVID-19 pandemic on people who experience imprisonment**

| Study | Was the sample frame appropriate to address the target population? | Were study participants recruited in an appropriate way? | Was the sample size adequate? | Were the study subjects and setting described in detail? | Was data analysis conducted with sufficient coverage of the identified sample? | Were valid methods used for the identification of the condition? | Was the condition measured in a standard, reliable way for all participants? | Was there appropriate statistical analysis? | Was the response rate adequate, and if not, was the low response rate managed appropriately? | No in one or more domains | Unclear in one or more domains |
| --- | --- | --- | --- | --- | --- | --- | --- | --- | --- | --- | --- |
| Altibi 2021 | yes | N/A | yes | yes | yes | yes | yes | yes | N/A |  |  |
| Bandara 2020 | yes | yes | yes | yes | unclear | no | unclear | no | yes | yes | yes |
| Berk 2021 | yes | N/A | yes | no | yes | yes | yes | yes | N/A | yes |  |
| Blair 2021 | yes | N/A | yes | no | N/A | yes | yes | yes | N/A | yes |  |
| Brinkley-Rubinstein 2021 | yes | N/A | yes | no | yes | yes | yes | yes | N/A | yes |  |
| Cerrato 2021 | N/A | N/A | yes | yes | N/A | yes | yes | yes | N/A |  |  |
| Chan 2021 | yes | N/A | yes | yes | yes | yes | yes | yes | N/A |  |  |
| Chin 2021[32] | yes | N/A | yes | yes | N/A | yes | yes | yes | N/A |  |  |
| Chin 2021[33] | yes | N/A | yes | yes | N/A | yes | yes | yes | N/A |  |  |
| Chin 2022 | yes | N/A | yes | yes | N/A | yes | yes | yes | N/A |  |  |
| Coleman 2020 | N/A | N/A | N/A | yes | N/A | yes | yes | yes | N/A |  |  |
| Collica-Cox 2020 | N/A | N/A | N/A | yes | N/A | yes | yes | yes | N/A |  |  |
| Crispim 2021 | yes | N/A | yes | yes | N/A | yes | yes | yes | N/A |  |  |
| Dunne 2021 | N/A | N/A | N/A | no | N/A | yes | yes | yes | N/A | yes |  |
| Getaz 2021 | yes | N/A | yes | no | yes | yes | yes | yes | N/A | yes |  |
| Giuliani 2021[25] | yes | N/A | yes | no | N/A | yes | yes | yes | N/A | yes |  |
| Giuliani 2021[26] | N/A | N/A | N/A | yes | N/A | yes | yes | yes | N/A |  |  |
| Gouvea-Reis 2021[23] | N/A | N/A | N/A | no | N/A | yes | yes | yes | N/A | yes |  |
| Gouvea-Reis 2021[24] | N/A | N/A | N/A | no | N/A | yes | yes | yes | N/A | yes |  |
| Hagan 2020[45] | yes | yes | yes | no | yes | yes | yes | yes | yes | yes |  |
| Hagan 2021[34] | yes | N/A | yes | yes | yes | yes | yes | yes | N/A |  |  |
| Hagan 2021[35] | N/A | N/A | N/A | yes | N/A | yes | yes | yes | N/A |  |  |
| Hershow 2021 | N/A | N/A | N/A | no | N/A | yes | yes | yes | N/A | yes |  |
| Jimenez 2020 | yes | N/A | yes | no | N/A | yes | yes | yes | yes | yes |  |
| Kennedy 2020 | yes | N/A | yes | no | N/A | yes | yes | yes | N/A | yes |  |
| Khairat 2021 | yes | yes | yes | yes | yes | yes | yes | yes | no | yes |  |
| KhudaBukhsh 2021 | N/A | N/A | yes | no | N/A | yes | yes | yes | N/A | yes |  |
| Lehnertz 2021 | N/A | N/A | N/A | no | N/A | yes | yes | yes | N/A | yes |  |
| Leibowitz 2021 | yes | N/A | yes | no | N/A | yes | yes | yes | N/A | yes |  |
| Lemasters 2020 | yes | N/A | yes | no | N/A | yes | yes | yes | N/A | yes |  |
| Lewis 2021 | N/A | N/A | N/A | no | N/A | yes | yes | yes | N/A | yes |  |
| Maner 2021 | yes | N/A | yes | no | N/A | yes | no | yes | N/A | yes |  |
| Marco 2020[27] | yes | N/A | N/A | yes | N/A | yes | yes | yes | N/A |  |  |
| Marco 2021[28] | N/A | N/A | N/A | yes | N/A | yes | yes | yes | N/A |  |  |
| Marquez 2021[36] | yes | N/A | yes | yes | N/A | yes | yes | yes | N/A |  |  |
| Marquez 2021[37] | yes | N/A | yes | yes | N/A | yes | yes | yes | N/A |  |  |
| Montanari 2021 | yes | unclear | unclear | no | yes | unclear | unclear | yes | unclear | yes | yes |
| Njuguna 2020 | N/A | N/A | N/A | yes | N/A | yes | yes | yes | N/A |  |  |
| Nowotny 2020 | yes | N/A | yes | no | N/A | yes | yes | yes | N/A | yes |  |
| Pettus-Davis 2021 | yes | yes | yes | yes | yes | yes | yes | yes | yes |  |  |
| Pocock 2020 | N/A | N/A | N/A | no | N/A | yes | unclear | yes | N/A | yes | yes |
| Puglisi 2021 | N/A | N/A | N/A | no | N/A | yes | yes | yes | N/A | yes |  |
| Ribeiro 2020 | yes | N/A | yes | no | N/A | yes | yes | yes | N/A | yes |  |
| Saloner 2020 | yes | N/A | yes | no | N/A | yes | yes | yes | N/A | yes |  |
| Stern 2021 | yes | yes | yes | yes | yes | yes | yes | yes | yes |  |  |
| Stufano 2021 | yes | yes | yes | yes | yes | yes | yes | yes | yes |  |  |
| Toblin 2021 | yes | N/A | yes | yes | N/A | yes | yes | yes | N/A |  |  |
| Tompkins 2021 | N/A | N/A | N/A | yes | N/A | yes | yes | yes | N/A |  |  |
| Vest 2021 | yes | N/A | yes | no | N/A | yes | yes | yes | N/A | yes |  |
| Wadhwa 2021 | yes | yes | yes | yes | yes | yes | yes | yes | yes |  |  |
| Wallace 2020 | yes | N/A | yes | no | unclear | yes | yes | yes | yes | yes | yes |
| Wallace 2021 | N/A | N/A | N/A | yes | N/A | yes | yes | yes | N/A |  |  |
| Wilburn 2021 | N/A | N/A | N/A | yes | N/A | yes | yes | yes | N/A |  |  |
| Zawitz 2021 | N/A | N/A | N/A | no | N/A | yes | yes | yes | N/A | yes |  |
