# Supplementary material for: The health impacts of the COVID-19 pandemic on adults who experience imprisonment globally: A mixed methods systematic review
Source: PLoS One. 2022 May 20;17(5):e0268866. doi: 10.1371/journal.pone.0268866 (PMC9122186; doi:10.1371/journal.pone.0268866)
Supplement: S2 Table — (DOCX) [file pone.0268866.s002.docx]

**S2 Table. Risk of bias for qualitative studies included in a systematic review on the health impacts of the COVID-19 pandemic on people who experience imprisonment**

| Study | Is there congruity between the stated philosophical perspective and the research methodology? | Is there congruity between the research methodology and the research question or objectives? | Is there congruity between the research methodology and the methods used to collect data? | Is there congruity between the research methodology and the representation and analysis of data? | Is there congruity between the research methodology and the interpretation of results? | Is there a statement locating the researcher culturally or theoretically? | Is the influence of the researcher on the research, and vice- versa, addressed? | Are participants, and their voices, adequately represented? | Is the research ethical according to current criteria or, for recent studies, and is there evidence of ethical approval by an appropriate body? | Do the conclusions drawn in the research report flow from the analysis, or interpretation of the data? | No in one or more domains | Unclear in one or more domains |
| --- | --- | --- | --- | --- | --- | --- | --- | --- | --- | --- | --- | --- |
| Gray 2021 | unclear | yes | yes | yes | yes | no | no | yes | yes | yes | yes | yes |
| Maycock 2021[29] | yes | yes | yes | yes | yes | no | no | yes | yes | yes | yes | no |
| Maycock 2021[30] | yes | yes | yes | yes | yes | no | no | yes | yes | yes | yes | no |
| Maycock 2021[31] | unclear | yes | yes | yes | unclear | no | no | yes | yes | yes | yes | yes |
| McLeod 2021 | yes | yes | yes | yes | yes | yes | no | yes | yes | yes | yes | no |
| Pyrooz 2020 | unclear | unclear | unclear | unclear | unclear | no | no | yes | yes | yes | yes | yes |
| Sorge 2021 | unclear | yes | yes | yes | yes | no | no | yes | yes | yes | yes | yes |
| Suhomlinova 2021 | yes | yes | yes | yes | yes | no | yes | yes | yes | yes | yes | no |
