# Supplementary material for: The health impacts of the COVID-19 pandemic on adults who experience imprisonment globally: A mixed methods systematic review
Source: PLoS One. 2022 May 20;17(5):e0268866. doi: 10.1371/journal.pone.0268866 (PMC9122186; doi:10.1371/journal.pone.0268866)
Supplement: S1 File — (DOCX) [file pone.0268866.s003.docx]

**S1 File. Search strategy**

Databases:

Medline, PsycINFO, Embase, the Cochrane Library, Applied Social Sciences Index and Abstracts, Sociological Abstracts, Sociology Database, Coronavirus Research Database, CINAHL, ERIC, Proquest Dissertations and Theses, Web of Science, Scopus, and MedRxiv

**Ovid MEDLINE: Epub Ahead of Print, In-Process & Other Non-Indexed Citations, Ovid MEDLINE® Daily and Ovid MEDLINE® <1946-Present>**

1. Prisoners/

2. Criminals/

3. Prisons/

4. Juvenile Delinquency/ or Criminal Psychology/

5. (correctional or felon* or imprison* or incarcerat* or jail* or offend* or prison* or convict* or inmate* or penitentiar* or detention or detainee* or parole or criminals).mp.

6. (community reentry or ex-convict* or ex-inmate* or former convict* or former inmate* or former offender* or former prisoner* or formerly incarcerated or offender* reenter* or offender* reentry or offender* reintegrat* or offender* release or out of jail or parole* or postincarceration or post-incarceration or postprison or postrelease or post-release or prison to community or prison to society or prisoner* reenter* or prisoner* reentry or prisoner* reintegrat* or prisoner* release* or probation* or rearrest* or recidivis* or recividate* or reconvict* or re-imprison* or re-incarcerat* or release* from prison or re-offend* or return to communit* or reoffend*).mp.

7. 1 or 2 or 3 or 4 or 5 or 6

8. Covid 19

9. Coronavirus/

10. Pandemic/

11. Sars cov 2.mp.

12. Sars cov2 or Sarscov2.mp.

13. 8 or 9 or 10 or 11 or 12

14. 7 and 13

15. Limit 14 to yr = “2019 -Current”

16. Remove duplicates from 15

**PsycINFO through OVID**

1. (correctional or felon* or imprison* or incarcerat* or jail* or offend* or prison* or convict* or inmate* or penitentiar* or detention or detainee* or parole or criminals).mp.

2. (community reentry or ex-convict* or ex-inmate* or former convict* or former inmate* or former offender* or former prisoner* or formerly incarcerated or offender* reenter* or offender* reentry or offender* reintegrat* or offender* release or out of jail or parole* or postincarceration or post-incarceration or postprison or postrelease or post-release or prison to community or prison to society or prisoner* reenter* or prisoner* reentry or prisoner* reintegrat* or prisoner* release* or probation* or rearrest* or recidivis* or recividate* or reconvict* or re-imprison* or re-incarcerat* or release* from prison or re-offend* or return to communit* or reoffend*).mp.

3. exp Prisons/ or exp Prisoners/ or exp Recidivism/ or exp Criminal Rehabilitation/ or ex-offender.mp. or exp Criminals/ or exp Correctional Institutions/ or exp Incarceration/ or exp Parole/ or Juvenile Delinquency/ or Criminal Conviction/ or Criminal Record/

4. 1 or 2 or 3

5. Exp Coronavirus/ or exp pandemics/

6. (Sars cov 2 or sars cov2 or sarscov2 or covid* or coronavirus).mp.

7. 5 or 6

8. 4 and 7

9. Limit 9 to yr=”2019 -Current”

10. Remove duplicates from 8

**Embase**

1. Prison/ or Prisoner/

2. (correctional or felon* or imprison* or incarcerat* or jail* or offend* or prison* or convict* or inmate* or penitentiar* or detention or detainee* or parole or criminals).mp.

3. Offender/

4. (community reentry or ex-convict* or ex-inmate* or former convict* or former inmate* or former offender* or former prisoner* or formerly incarcerated or offender* reenter* or offender* reentry or offender* reintegrat* or offender* release or out of jail or parole* or postincarceration or post-incarceration or postprison or postrelease or post-release or prison to community or prison to society or prisoner* reenter* or prisoner* reentry or prisoner* reintegrat* or prisoner* release* or probation* or rearrest* or recidivis* or recividate* or reconvict* or re-imprison* or re-incarcerat* or release* from prison or re-offend* or return to communit* or reoffend*).mp.

5. 1 or 2 or 3 or 4

6. Coronavirinae/ or coronavirus infection/

7. Pandemic/

8. (Sars cov 2 or sars cov2 or sarscov2 or covid* or coronavirus).mp.

9. 6 or 7 or 8

10. 5 and 9

11. limit 10 to yr="2019 -Current"

12. Remove duplicates from 11

**The Cochrane Library:**

1. Title/Abstract/Keyword: (correctional or felon* or imprison* or incarcerat* or jail* or offend* or prison* or convict* or inmate* or penitentiar* or detention or detainee* or parole or criminals)

2. Title/Abstract/Keyword: ((covid) or (coronavirus) or (sars cov 2) or (sars cov2) or (sarscov2))

3. 1 AND 2

**EBSCOhost: Social Sciences Abstracts (1983-present), CINAHL (1981-present)**

1. (correctional or felon* or imprison* or incarcerat* or jail* or offend* or prison* or convict* or inmate* or penitentiar* or detention or detainee* or parole or criminals)

2. ((covid) or (coronavirus) or (sars cov 2) or (sars cov2) or (sarscov2))

3. Limit publication date: 2019-2021

**ERIC (1966-current), Proquest Dissertations and Theses A&I information, Applied Social Sciences Index & Abstracts (ASSIA) (1987-current), Sociological Abstracts (1952-current), Sociology Database (1985-current), Coronavirus Research Database, CINAHL**

1. (correctional or felon* or imprison* or incarcerat* or jail* or offend* or prison* or convict* or inmate* or penitentiar* or detention or detainee* or parole or criminals) in NOFT

2. ((covid) or (coronavirus) or (sars cov 2) or (sars cov2) or (sarscov2)) in NOFT

3. 1 AND 2

**WEB OF SCIENCE**

1. TS=(correctional or felon* or imprison* or incarcerat* or jail* or offend* or prison* or convict* or inmate* or penitentiar* or detention or detainee* or parole or criminals)

2. TS=((covid or coronavirus or “sars cov 2” or “sars cov2” or sarscov2))

3. 1 AND 2

**Scopus**

1. TITLE-ABS-KEY (correctional or felon* or imprison* or incarcerat* or jail* or offend* or prison* or convict* or inmate* or penitentiar* or detention or detainee* or parole or criminals)

2. TITLE-ABS-KEY ((covid) OR (coronavirus) OR ("sars cov 2") OR (“sars cov2”) OR (sarscov2)) AND PUBYEAR > 2018
