# Supplementary material for: The health impacts of the COVID-19 pandemic on adults who experience imprisonment globally: A mixed methods systematic review
Source: PLoS One. 2022 May 20;17(5):e0268866. doi: 10.1371/journal.pone.0268866 (PMC9122186; doi:10.1371/journal.pone.0268866)
Supplement: Attachment — Submitted filename: Reviewer feedback PONE-D-22-05150.docx [file pone.0268866.s005.docx]

**Summary**

Thank you for taking the time to conduct a thoughtful and careful review of the current published/pre-printed literature on COVID-19’s impact on those who have been incarcerated; this is crucial and important work! The methodological approach was transparent and sound, and I applaud the authors for their diligent work. Because the purpose of this article is a review, I believe that there are substantial revisions that are necessary for this to sufficiently serve that purpose to readers. It’s clear that there was a lot of data extraction done, and this is fantastic, but there was minimal synthesis, contextualization, and organization of these findings, which is crucial for providing a thorough and manageable review of literature. While suggested revisions (particularly for the data synthesis and discussion sections) are considerable, much of the content is already in the paper, but could use some reorganizing, reframing, or a deeper synthesis. As you aptly highlighted, there are more studies that will be coming out and more work in this area that will need to be done, and therefore, providing more clear recommendations based on the large breadth of information reviewed (taking into account the sampled studies’ limitations,) would be a wonderful addition to help drive home the value of all this great work. More detailed and specific comments are provided below – thank you for allowing me the opportunity to read your paper, and I hope these comments prove useful to you!

**Introduction – Comments**

- This section was compelling, concise, well-framed! Below are just a few comments on some areas to further strengthen this section:
  - I think some clarification around the risk of COVID-19 being introduced into prisons being high could be useful here. The criminal justice system (including courts and prisons) altered its practices considerably throughout the pandemic, and so acknowledging that even in the presence of substantial changes and policy shifts (e.g., prisons massively changing how/when people moved, implementing lockdowns, etc.), it was still impossible to effectively halt the introduction of COVID-19 into prisons.
  - Clarifying point about addressing increases in drug supply is advised, (e.g., that this is specific to individuals upon release.) If you want to address use in prison more explicitly, suggesting the potential for an increase in relapses (or new use) would be warranted and follows from the statement about worsened mental health for those who have been incarcerated during the pandemic.

**Eligibility Criteria – Comments**

- This section was thorough and clear, and your methodological approach was sound! Your clarity and transparency in how use language is especially appreciated! I want to especially thank you for bringing up the point about policies not necessarily aligning with how prisons are run in practice! Below are just a few comments on some areas to further strengthen this section:
  - Just highlighting a really small typo (nothing major): “The first criterion was a focus adults…”

**Characteristics of Included Studies – Comments**

- Table 1 is incredibly helpful and a great addition to this paper! Below are just a few comments on some areas to further strengthen this section:
  - While this is only a very minor point, if the table was created in such a way that allows you to easily resort it, that might be very helpful for readers to understand the distribution of studies in your overall sample more easily. I think either resorting based on either time or study location could be particularly helpful.

**Risk of bias – Comments**

- Thank you for your transparency in addressing the quality of the studies included! Elaborating on the domains where quality indicators were not met would be helpful (e.g., do they all tend to be different, or are there patterns amongst which indicators are not met?) Knowing this information would be helpful for informing the interpretation of results.

**Data Synthesis – Burden of COVID infection – Comments**

- This section is very thorough! However, I found myself getting very bogged down by a lot of numbers and felt that synthesis, organization, and interpretation of findings was lacking. I also found myself having trouble with interpretation of these figures given the information about study quality (i.e., might the bias be impacting reported figures, and if so, do we expect these numbers to be under/over-estimates? Or maybe not representative?) Below are some specific comments on potential routes to help strengthen this section:
  - There are a lot of different numbers for incidence rates/percent positivity presented in this section, which is excellent, but I found myself getting a bit bogged down in the details. Graphs here I think could be particularly helpful and given the fact that the number of figures/tables are not limited, this is strongly advised for helping readers be able to make sense of all of these figures. Then, the written sections can focus more heavily on synthesis. For example, instead of listing something like “Study *A* found Incident Rate *A* in Time *A* and Location *A*,” starting with something like, “The range in cumulative incidence rates ranged from *x* to *y*, with the lowest being in (Time *B*/Location *C*, etc.)”
  - For the figures, even if it’s just a bar graph, ordered by date (when available) with location-specific details included (and patterns/colors associated with location potentially,) with both prison-specific and overall population-specific side-by-side bars. Then the text can focus on describing how much higher these figures were in-prison, compared to respective populations, over time/space. That or a table could be helpful!
  - As mentioned above, quality of studies needs to be explicitly woven into interpretation so that we can more accurately and appropriately interpret the figures reported from these studies. I do not think this necessarily means calling out the studies where quality indicators were not met.

**Discussion – Comments**

- I appreciated the transparency about how early this study is, as this was important to acknowledge, and is helpful! In this section, I was really hoping for a lot more in terms of synthesis, recommendations, and limitations. Below are a few more specific suggestions that may help support this section:
  - Limitations: I was hoping to see more discussion of what seemed to be some geographic-specific differences in the types of studies conducted. For example, the U.S. seemed to focus a lot more on the epidemiological data (e.g., incidence rates, percent positivity, etc.), while data outside of the U.S. – namely Europe – seemed to be the primary source of qualitative data. This is crucial, and likely reflects considerable differences in penological culture.
  - I would love to see more on recommendations! You (rightly!) suggest individuals who are incarcerated need to be prioritized in handling COVID-19 and other public health emergencies. How do we accomplish this? What future studies need to be conducted, based off of what you found? Did it seem that some places were doing better than others in terms of reducing the burden of COVID-19 on its incarcerated populations? Even just highlighting this is important – more work can be done in future studies that could illuminate why this might have been the case. Especially in regards to mental health of residents in prisons – what can be done to support these individuals while still keeping their risk of infection as low as possible (e.g., increasing access to tablets/phone calls, etc.)?
