# Supplementary material for: The health impacts of the COVID-19 pandemic on adults who experience imprisonment globally: A mixed methods systematic review
Source: PLoS One. 2022 May 20;17(5):e0268866. doi: 10.1371/journal.pone.0268866 (PMC9122186; doi:10.1371/journal.pone.0268866)
Supplement: Attachment — Submitted filename: Response to reviewers_May 4 2022.docx [file pone.0268866.s006.docx]

**Response to reviewers**

*Journal Requirements:

Please review your reference list to ensure that it is complete and correct. If you have cited papers that have been retracted, please include the rationale for doing so in the manuscript text, or remove these references and replace them with relevant current references. Any changes to the reference list should be mentioned in the rebuttal letter that accompanies your revised manuscript. If you need to cite a retracted article, indicate the article’s retracted status in the References list and also include a citation and full reference for the retraction notice.*

We have reviewed the references and have made minor edits to the list of references. We have not added or removed any references.

*Reviewers' comments:*

***Comments to the Author*** *1. If the authors have adequately addressed your comments raised in a previous round of review and you feel that this manuscript is now acceptable for publication, you may indicate that here to bypass the “Comments to the Author” section, enter your conflict of interest statement in the “Confidential to Editor” section, and submit your "Accept" recommendation.*

*Reviewer #1: All comments have been addressed*

*Reviewer #2: All comments have been addressed*

*2. Is the manuscript technically sound, and do the data support the conclusions?

The manuscript must describe a technically sound piece of scientific research with data that supports the conclusions. Experiments must have been conducted rigorously, with appropriate controls, replication, and sample sizes. The conclusions must be drawn appropriately based on the data presented.*

*Reviewer #1: Yes*

*Reviewer #2: Yes*

*3. Has the statistical analysis been performed appropriately and rigorously?*

*Reviewer #1: N/A*

*Reviewer #2: N/A*

*4. Have the authors made all data underlying the findings in their manuscript fully available?

The [PLOS Data policy](http://www.plosone.org/static/policies.action#sharing) requires authors to make all data underlying the findings described in their manuscript fully available without restriction, with rare exception (please refer to the Data Availability Statement in the manuscript PDF file). The data should be provided as part of the manuscript or its supporting information, or deposited to a public repository. For example, in addition to summary statistics, the data points behind means, medians and variance measures should be available. If there are restrictions on publicly sharing data—e.g. participant privacy or use of data from a third party—those must be specified.*

*Reviewer #1: (No Response)*

*Reviewer #2: Yes*

*5. Is the manuscript presented in an intelligible fashion and written in standard English?

PLOS ONE does not copyedit accepted manuscripts, so the language in submitted articles must be clear, correct, and unambiguous. Any typographical or grammatical errors should be corrected at revision, so please note any specific errors here.*

*Reviewer #1: Yes*

*Reviewer #2: Yes*

*6. Review Comments to the Author

Please use the space provided to explain your answers to the questions above. You may also include additional comments for the author, including concerns about dual publication, research ethics, or publication ethics. (Please upload your review as an attachment if it exceeds 20,000 characters)*

*Reviewer #1: Thank you for revising your manuscript for review! This is important work, and I believe this paper will be helpful for highlighting how imperative it is to better understand the impact of public health emergencies in the carceral context. 
I believe the authors have done an excellent job on the revision, with tables and figures that helpfully summarize the information and make the manuscript's content more accessible to readers. Limitations and interpretation were bolstered to help address future outlets for research and the limits to what was available at the time of this synthesis.*

We appreciate these comments. *I think the Discussion section may still benefit from small edits, namely a stronger stance on the necessity of future research. The authors appropriately identify that there are a variety of reasons that are likely (individually, but also collectively,) contributing to the wide variation in COVID-19 positivity rates and other factors related to the spread of the virus. As the authors identified, published studies may have a unique influence on public policy, and therefore, given their manuscript will hopefully be published (as I will recommend here,) developing this section and more concretely suggesting future avenues could be particularly useful.

Specifically, identifying how to tease out the differences and contributing factors is crucial to understanding the toll of the virus and how to alleviate the burden of health effects on incarcerated populations. The Discussion talks about potential follow-ups as valuable, though they are necessary given the appropriately strong final recommendation that incarcerated individuals need to be thoughtfully considered and properly supported, particularly during public health emergencies.*

Thank you for this suggestion. We have elaborated on the content about future research in the fifth paragraph of the Discussion. *In all, I am thankful to the authors for the manuscript and the valuable summary of literature and will recommend its publication. Thank you!*

Thank you.

*Reviewer #2: Thank you for your thoughtful responses and addressing of comments. I believe it is a very strong manuscript with critical and timely information for researchers, policymakers, and people living and working in prisons, and have no further feedback.*

Thank you.
